# From Design to Accountable Impact for Data Dashboards in Health Care

**DOI:** 10.2196/98272

**Published:** 2026-05-25

**Authors:** Barbara-Jo Achuff

**Affiliations:** 1 Department of Pediatrics, Department of Biomedical Informatics, Vanderbilt School of Medicine, Vanderbilt University Medical Center, 2200 Children's Way, 5121 DOT, Nashville, TN, 37232, United States, 1 615-343-2996

**Keywords:** health care dashboards, data visualization, visual analytics, clinical decision support, workflow integration, data governance, data quality, implementation science, quality improvement

## Abstract

Vornhagen and colleagues synthesize design practices for the development of health care dashboards and provide a timely reference for a rapidly expanding class of tools that increasingly mediate clinical decisions and quality improvement. The authors have defined 4 pillars of design with associated practices, establishing a practical approach to the thoughtful development of useful dashboards. Building on these pillars, this commentary proposes accountable dashboarding, defined as making explicit the causal chain linking data sources and governance to visualization, interpretation, action, and measurable outcomes. Dashboards could be treated as sociotechnical interventions in which upstream choices are inseparable from the user interface and downstream decision-making. Evaluation should extend beyond usability and satisfaction to include decision quality and behavioral proxies, unintended consequences, and patient-centered outcomes of dashboard-informed interventions.

## Design Practices for Data Dashboards

The scoping review by Vornhagen and colleagues [1] is a welcome synthesis of dashboard design practices in health care, drawing together guidance scattered across visualization, human factors, clinical informatics, and quality improvement literature. Their work is timely: dashboards now span bedside decision support, unit-level operations, and longitudinal quality surveillance, often built ad hoc under real-world constraints and evaluated inconsistently.

At the same time, the heterogeneity captured in a scoping review can be a double-edged sword. Implementers may adopt design practices as a checklist of features without clarity about which practices matter for which settings, workflows, and outcomes. As dashboards increasingly influence clinical decisions and resource allocation, the field should move toward accountable dashboarding, where dashboards are designed, implemented, and reported as interventions with explicit mechanisms and measurable effects.

## Design Is Upstream of the Interface: Dashboards Are Sociotechnical Systems

Vornhagen and colleagues [1] describe themes emerging from the review’s secondary analysis and group them into pillars. The first, pillar 1, includes the design approach and planning work that precedes dashboard building and deployment. A dashboard’s most consequential design choices often occur before the interface is built and are intimately tied to the other themes, or pillars. Metric definitions, data provenance, missingness, refresh cadence, and transformation logic determine whether clinicians trust what they see and whether the dashboard fits the decision tempo of care. In practice, users experience these upstream elements as part of the product because they determine timeliness, stability, and credibility, core attributes of Tufte’s “clear thinking made visible” in information design [2].

Building on this first pillar, this commentary suggests that accountable dashboarding requires describing sociotechnical components as first-class design elements, including metric governance and versioning, which concerns who owns measures and how changes are updated and communicated (ie, control and governance); data provenance and quality, which encompasses the source systems that feed the dashboard, known gaps, and the handling of missing or delayed data (ie, truth and veracity); alingment of refresh cadence to decision moments (ie, fidelity and alignment); and workflow integration and role specificity, which refers to where dashboarding is used (eg, prerounds, rounds, huddles, or quality improvement meetings) and what actions should follow (ie, usability, orchestration, and integration). These are not merely technical details; they can shape behavior, adoption, and sustainability and are essential for others to interpret results and reproduce success.

## Linking Design to Outcomes: Evaluation Should Match the Stakes

Vornhagen and colleagues [1] highlight evaluation approaches found in the literature and note that dashboard evaluation too often ends at usability or satisfaction. Those outcomes matter but may be insufficient for tools intended to change behavior and improve outcomes; usability principles remain necessary but not definitive evidence of effectiveness [3]. A usable dashboard can be clinically inert; conversely, a clinically valuable dashboard can fail if it increases workload or creates new sources of error.

A practical next step could include a required simple logic model that follows an evaluation chain of (1) data, (2) visualization, (3) interpretation, (4) decision and action, and (5) outcome. At a minimum, evaluation reports need to be developed and should include adoption and reach (who used it, how often, and in which workflow moments); behavioral proxies (changes in ordering, documentation, protocol adherence, or other care processes); workload and unintended consequences (time cost, duplication, workarounds); outcomes (patient-centered outcomes where feasible, otherwise tightly linked intermediate outcomes); and sustainability (maintenance burden and performance over time, including measure drift).

Numerous methods exist to measure and improve health care quality, safety, and value. By providing a structured framework, implementation science and quality improvement reporting guidelines enhance the rigor of these efforts without requiring new trials in every setting [4-6].

## Bedside and Longitudinal Value: Lessons From a Pediatric Intensive Care Unit

In our tertiary academic pediatric intensive care unit, the need arose to make sedation medication exposure visible at both the bedside and unit level because existing electronic record views were difficult to interpret longitudinally and did not reliably support practice standardization. A dashboard that refreshed daily was developed with multidisciplinary stewardship using electronic medication administration record data to visualize sedation exposure for intensive care unit patients in two ways: (1) patient-level longitudinal profiles across hospitalization and/or postoperative day, and (2) aggregate unit-level trends over months for quality metrics. To make data interpretable and comparable, upstream transformation logic (eg, conversion to standardized opioid and benzodiazepine equivalents and calculation rules for continuous infusions) was treated as a core design component.

In practice, the dashboard enabled the identification of improvable patterns at scale, including dosing variation and spikes at specific hours—signals difficult to detect using static reports or manual review—and supported integration into bedside rounds and ordering behaviors. The dashboard was also used to monitor practice after the implementation of standardized sedation assessment and education. Over time, benzodiazepine exposure trended downward with attenuation of day-and-night variation. Although these changes occurred within a multicomponent improvement effort, the dashboard functioned as both a diagnostic tool (identifying targets) and a sustainability tool (monitoring after change). We identified that a more frequent refresh could clinically support more timely clinical discussions and action. This dual role, supporting bedside discussion while enabling longitudinal surveillance, may be underemphasized in the dashboard design literature and deserves explicit reporting and evaluation.

## Equity and Accessibility Should Be Baseline Requirements

A persistent gap in dashboard development is that equity and accessibility are treated as optional enhancements rather than requirements. Dashboards influence attention, prioritization, and standardization, and can differentially affect patient subgroups and clinical roles. Three baseline commitments are pragmatic and feasible: accessibility by default using design informed by the Web Content Accessibility Guidelines (contrast, typography, and color choices accommodating color vision deficiencies) [7], role-specific usability testing across nurses, pharmacists, trainees, and attendings, and lastly, equity-sensitive evaluation to assess whether dashboard-driven metrics or interventions perform differently across patient subgroups or reflect documentation bias [8].

## Extending the Pillars

Vornhagen and colleagues [1] should be congratulated for providing a strong foundation in their scoping review. To enhance generalizability, dashboard research and implementation reports should add three expectations: (1) publish the logic model and the moment of use, defined as when and where in the real workflow the dashboard is accessed and what action it is intended to trigger; (2) report governance and data-quality processes as part of design (sources, refresh cadence, missingness handling, and metric ownership and versioning); and (3) adopt a minimum evaluation and reporting set combining usability with adoption, workflow burden, unintended consequences, sustainability, outcomes, and equity and accessibility checks.

Dashboards are now part of the clinical infrastructure. The goal is not only to design them well, but to make mechanisms and impacts knowable so others can reproduce benefits, avoid predictable failures, and ensure improvements are trustworthy and equitable.
